# Selection on the *Drosophila* seminal fluid protein Acp62F

**DOI:** 10.1002/ece3.605

**Published:** 2013-05-23

**Authors:** Alex Wong, Howard Rundle

**Affiliations:** 1 Department of Biology, Carleton UniversityOttawa, Canada; 2 Department of Biology and Center for Advanced Research in Environmental Genomics, University of OttawaOttawa, Canada

**Keywords:** Experimental evolution, natural selection, protease inhibitor, sexual selection, sperm competition

## Abstract

Sperm competition and sexual conflict are thought to underlie the rapid evolution of reproductive proteins in many taxa. While comparative data are generally consistent with these hypotheses, few manipulative tests have been conducted and those that have provided contradictory results in some cases. Here, we use both comparative and experimental techniques to investigate the evolution of the *Drosophila melanogaster* seminal fluid protein Acp62F, a protease inhibitor for which extensive functional tests have yielded ambiguous results. Using between-species sequence comparisons, we show that Acp62F has been subject to recurrent positive selection. In addition, we experimentally evolved populations polymorphic for an *Acp62F* null allele over eight generations, manipulating the opportunities for natural and sexual selection. We found that the *Acp62F* null allele increased in frequency in the presence of natural selection, with no effect of sexual selection.

## Introduction

In a broad range of taxa including vertebrates, invertebrates, fungi, and plants, some of the most rapidly evolving proteins encoded in the genome are those contributing to reproductive success (for reviews, see Clark et al. 2006; Chapman 2008; Wong 2011). In *Drosophila*, for example, seminal fluid proteins (SFPs) that are transferred from males to females during copulation diverge quickly at the sequence level, and the proportion of SFP-encoding genes subject to positive selection is unusually high compared with the rest of the genome (e.g., Civetta and Singh 1995; Haerty et al. 2007). Moreover, the complement of SFPs produced by different species can vary drastically, with apparently high rates of gene loss and recruitment of new genes (e.g., Holloway and Begun 2004; Mueller et al. 2005; Wagstaff and Begun 2005a, 2007; Haerty et al. 2007; Findlay et al. 2008; Kelleher et al. 2009).

Several hypotheses have been proposed to explain the rapid evolution of reproductive proteins. In internally fertilizing animals, a leading proposal is that postcopulatory sexual selection is responsible, for example, via sperm competition or sexual conflict. Sperm competition arises when sperm from multiple males are present at the same time in the reproductive tract of a female. If an SFP variant leads to an increased paternity share, then that variant should be favored by sexual selection. Consistent with an effect of sperm competition on SFP evolution, several rapidly evolving *Drosophila melanogaster* SFP genes have known effects on sperm storage and/or sperm competition (Acp36DE: Neubaum and Wolfner 1999; Chapman et al. 2000; Acp29AB: Wong et al. 2008; CG9997: Ram and Wolfner 2007).

Similarly, a number of *Drosophila* SFPs are known to have effects on processes potentially involved in interlocus sexual conflict. Interlocus sexual conflict arises when the optimal outcome of an interaction between the sexes is different for males and females. For example, females of some species may gain by mating with multiple males (Simmons 2005) owing to sperm depletion (e.g., Thornhill and Alcock 1983), sperm quality (e.g., Keller and Reeve 1995), and other indirect benefits, and/or from direct benefits such as nuptial gifts (e.g., Simmons et al. 1999). By contrast, it is typically in a male's interest that his partner does not mate with other males. As such, the optimal remating rate may be higher for females than it is for males (e.g., Gavrilets and Hayashi 2006). In *D. melanogaster*, the “sex-peptide (SP) network” of proteins modulates remating rate, as well as several other postmating responses (Chapman et al. 2003; Liu and Kubli 2003; Ram and Wolfner 2009; LaFlamme et al. 2012), and at least one member of the network has been subject to positive selection (CG9997; Wong et al. 2012). The SP network may in fact be involved in sexual conflict in several different ways; in addition to its effects on female remating, a functional SP network is necessary for the mating-induced reduction in female life span observed in this species (Wigby and Chapman 2005).

Consistent with the hypothesis that sexual selection is a primary cause of rapid reproductive protein evolution, comparative studies indicate a correlation between mating system and the rates of evolution of some reproductive proteins, including SFPs. That is, for some individual proteins, rates of evolution are higher in polyandrous lineages than in monandrous lineages, where postcopulatory sexual selection is less likely to occur (e.g., Ramm et al. 2008; Finn and Civetta 2010; Prothmann et al. 2012). Moreover, average rates of reproductive protein evolution are higher in polyandrous lineages in comparison with monandrous lineages in primates and in *Drosophila* (Wagstaff and Begun 2005b, 2007; Kelleher et al. 2007; Almeida and Desalle 2009), again suggesting that sexual conflict is important in the evolution of reproductive proteins. Additional sequence-based evidence for an effect of sexual selection on rates of reproductive protein evolution comes from Clark et al. (2009), who showed strong linkage disequilibrium between the abalone sperm protein lysin and its egg-receptor vitteline envelope receptor for lysin, as predicted under models of sexual selection.

In this study, we investigated the evolution of the *D. melanogaster* SFP Acp62F. Acp62F has been extensively studied using genetic and biochemical methods, but its function nevertheless remains unclear. The Acp62F protein is a protease inhibitor (Lung et al. 2002), a biochemical characteristic that it shares with numerous other SFPs (Mueller et al. 2004; Laflamme and Wolfner 2012). Overexpression of Acp62F in larvae or adults is toxic (Lung et al. 2002; Mueller et al. 2007), and a QTL containing the *Acp62F* locus is associated with postmating female mortality in interspecific introgression lines (Civetta et al. 2005), suggesting that this protein may be involved in seminal fluid toxicity. If so, then one would expect this protein to have other fitness-enhancing effects that outweigh its costs to females (unless toxicity is itself beneficial to males – see Johnstone and Keller 2000). Such a benefit has not been identified; indeed, Mueller et al. (2008) found that *Acp62F* knockout males performed *better* in sperm competition than did wild-type (wt) males. Mueller et al. (2008) also showed that Acp62F is required for normal proteolytic cleavage of the egg-laying hormone Ovulin, but the functional consequences of this phenotype are unclear as mates of *Acp62F* knockout males did not show any obvious defect in egg laying.

Here, we use computational and manipulative approaches to investigate the nature of selection acting on Acp62F. Using comparative sequence analyses, we test for a signature of positive selection on Acp62F. Furthermore, in a two-way factorial evolution experiment, we independently manipulate the opportunities for selection arising from variation in sexual and nonsexual fitness in replicate experimental populations polymorphic for *Acp62F* wt and null alleles, and then determine the consequences of this manipulation by tracking changes across generations in allele frequencies. This approach allows the net effects of Acp62F on sexual and nonsexual fitness to be integrated by the evolutionary process itself.

## Materials and Methods

### Inference of selection from comparative sequence data

We obtained nucleotide sequences of Acp62F from *D. melanogaster*, and orthologous sequences from *Drosophila simulans*, *Drosophila sechellia*, *Drosophila yakuba,* and *Drosophila erecta* from FlyBase (FlyBase IDs: FBgn0020509, FBgn0043400, FBgn0069552, FBgn0107140, FBgn0237654). Translated sequences were aligned using Muscle (Edgar 2004) and back translated to nucleotide sequences using T-COFFEE (Notredame et al. 2000). The resulting multiple sequence alignment is included in Appendix 1.

We used three methods to infer recurrent positive selection on Acp62F. The first method implements the “sites” models in codeml, part of the PAML package (Yang 2007). Findlay et al. (2008) conducted the same analysis; we replicate it here to confirm their results. The sites models allow variation in ω (*d*N/*d*S) between different codons within a gene, but assume that all lineages experience the same distribution of ω. Two null models were used, M7 and M8A (Yang et al. 2000), each of which restricts ω to be less than or equal to one, thus disallowing positive selection. M7 assumes a beta-distribution for ω and M8A assumes a beta-distribution as well as an extra class of sites in which ω = 1. The alternative model M8 assumes a beta-distribution for ω (restricted to be ≤1), but adds a class of sites with ω ≥ 1. M8 can be compared with either null model via a likelihood ratio test (with 2 and 1 df for M7 and M8A, respectively), and a significant rejection of the null model is evidence in favor of positive selection on a subset of codons.

We additionally used the random-effects likelihood (REL) and fixed-effects likelihood (FEL) methods of Kosakovsky Pond and Frost (2005) as more robust tests for positive selection on Acp62F. REL and FEL analyses were performed on the hypothesis testing using phylogenies datamonkey server (Pond and Frost 2005). These methods allow variation in both *d*N and *d*S, whereas the models implemented in PAML assume a single value of *d*S. REL assumes predefined distributions for *d*N and *d*S, and after initial inference of parameter values uses an empirical Bayes approach to infer selection on each site. As such, like PAML, the REL analysis assigns to each codon a posterior probability that it is under positive selection, with higher posterior probabilities indicating greater confidence that selection operates on the given codon. FEL, in contrast, directly estimates *d*N and *d*S at each site. Simulation results suggest that REL may be subject to higher false-positive rates for alignments with few species, such as the five-species alignment used here, whereas FEL does not seem to suffer from this problem (Kosakovsky Pond and Frost 2005). For each codon, FEL estimates the probability of obtaining the observed *d*N and *d*S values under a neutral model (i.e., a *P*-value under neutrality), with lower *P*-values indicating rejection of neutrality in favor of positive selection.

### Experimental evolution of populations polymorphic for an Acp62F null allele

An inbred stock of *D. melanogaster* homozygous for an *Acp62F* null allele (*ΔAcp62F*) on a *w*^*1118*^ background, as well as a genotype-matched *w*^*1118*^ *wt* allele, was kindly provided by N. Buehner and M. F. Wolfner. The *ΔAcp62F* allele was previously described by Mueller et al. (2008), and was constructed using the precise gene-targeting method of Rong and Golic (2000). We confirmed the *Acp62F* genotypes of both the *wt* and *ΔAcp62F* lines prior to the beginning of the experiment. All populations were maintained on a 12-h light:dark cycle on cornmeal food at 25°C.

Twelve replicate populations were maintained throughout the course of the experiment. Each population was started with the *Acp62F* null and wt alleles each at a frequency of 0.5, with genotypes at Hardy–Weinberg equilibrium. Three populations were assigned to each of the four possible treatments in a factorial design following Rundle et al. (2006): natural and sexual selection both present (**NS**), natural selection present and sexual selection greatly reduced (**Nx**), natural selection greatly reduced and sexual selection present (**xS**), or natural and sexual selection both reduced (**xx**). In populations in which sexual selection was reduced, a single sexually mature virgin female and a single mature male (both aged 3–4 days) were placed in a vial together for 2–3 days to mate. In populations in which sexual selection was present, a single virgin female was housed with 3–5 males for the same period of time for mating, thus allowing both pre- and postcopulatory sexual selection. After mating, males were discarded and 50 females per population were individually placed in separate vials to lay eggs for 2–3 days, after which the females were frozen at −80°C. For populations in which natural selection was reduced, each female contributed one female offspring and one (sexual selection reduced) or four (sexual selection present) male offspring to the next generation. For natural selection present treatments, all offspring produced by all females were pooled and 50 females and 50 (or 200) males were randomly selected for the next generation, such that each female contributed to the next generation in proportion to her productivity. The natural selection treatment thus incorporated virtually all nonsexual components of selection, including female fecundity, male fecundity, and egg-adult survival of offspring.

### Genotyping

DNA was extracted from individual frozen females at generations 6 and 8 following the method of Gloor et al. (1993). Single flies were ground in 50 μL of squish buffer (10 mmol/L Tris-Cl pH 8.2, 1 mmol/L EDTA, 25 mmol/L NaCl, and 200 μg/mL proteinase K) and incubated for 1 h at 37°C. Proteinase K was then inactivated at 95°C for 5 min, samples were centrifuged, and the supernatant was used as a template for subsequent polymerase chain reaction (PCR) reactions.

By PCR 12–15 individuals were genotyped from each population at generation 6 and at generation 8. Two primer pairs were used: primer pair 1 (Acp62F-screen1 and Acp62F-screen2 from Mueller et al. 2008) consisted of primers flanking the *Acp62F* locus, and in principle should generate a long (1635 bp) PCR product from the *wt* allele, and a short (825 bp) allele from the *ΔAcp62F* allele. However, we found that amplification of the long *wt* allele was inconsistent, particularly in heterozygotes. Thus, we additionally used primer pair 2 (Acp62F-4: ACTGGGCAGCAGGTGGAATG; Acp62F-5: CGAACTTTAAGTGCTTTAGCAG), consisting of one primer within the *Acp62F* gene, and one in the downstream flank. This primer pair consistently amplified a 275 bp fragment from the wt allele, and produced no product from the knockout allele. All samples were genotyped twice with each primer pair to ensure reproducibility, and samples producing no PCR product with either primer pair were discarded. Remaining individuals were scored as homozygous for either the null or *wt Acp62F* allele, or as heterozygotes. Raw genotype data are provided in Appendix 2.

### Statistical analyses

Counts of the *Acp62F* wt and null alleles for each population sample were calculated from the genotype data. Effects of natural selection, sexual selection, and their interaction on allele counts were assessed via binomial regression, using a call to the glm() function in R (R Core Development Team 2011).

## Results and Discussion

### Comparative evidence for positive selection on Acp62F

Population-genetic and comparative sequence analyses have documented positive selection on many genes encoding *Drosophila* SFPs (reviewed in Civetta 2003; Clark et al. 2006; Chapman 2008; Wong and Wolfner 2012). In keeping with this broad trend, we find evidence of positive selection on *Acp62F* (Tables 1 and 2) using sequence data from five *Drosophilid* species. Using the PAML package (Yang 2007), we find that the data fit a model (M8) that allows positive selection on a subset of codons significantly better than either of the two null models that do not allow positive selection. Under M8, 27% of codons fall into the positively selected class, with ω = 2.92. Analysis of the same dataset using the REL method of Kosakovsky Pond and Frost (2005), which is more robust to synonymous site rate variation, also provides evidence for positive selection (Table 2), with five codons inferred to have ω > 1 with a posterior probability of >0.9. Analysis using the FEL method (Kosakovsky Pond and Frost 2005) provides less convincing evidence of selection, with only one site showing marginal evidence for an elevated *d*N/*d*S ratio (site 45; *P* = 0.08; Table 2). This discrepancy may suggest that the limited number of species used here produces false positives with PAML and with the REL method; alternatively, there may be insufficient power to robustly infer selection using FEL.

**Table 1:** Statistical evidence for positive selection on *Acp62F* from PAML

Model	Log likelihood	−2ΔlnL versus M8	*P*-value	Proportion selected sites	ω
M8	−1044.84	NA	NA	0.27	2.92
M8A	−1051.78	13.88	1.9 × 10^−4^	NA	NA
M7	−1052.20	14.72	6.4 × 10^−4^	NA	NA

**Table 2:** Sites under positive selection under the codon models M8, FEL, and REL

Site	M8 posterior probability	REL posterior probability	FEL *P*-value
40	**0.93**	**0.96**	>0.5
42	**0.99**	**0.97**	0.13
45	**0.90**	**0.99**	0.08
99	**0.94**	**0.97**	0.30
100	**0.91**	<0.5	>0.5
103	**0.99**	0.62	>0.5
113	**0.95**	**0.96**	0.23
115	**0.99**	0.78	>0.5

For M8 and REL, posterior probabilities that a site is under positive selection are given, with values >0.9 highlighted in bold. For FEL, *P*-values give the probability that the site is *not* under positive selection, that is, the *P-*value under neutrality.

Acp62F is a known protease inhibitor (Lung et al. 2002), and knockout of *Acp62F* in males has been shown to slow the proteolysis of at least one other SFP in mated females (Mueller et al. 2008). Protease inhibition is thought to occur via mimicry of the target protease's normal substrate, with the inhibitor's reactive center loop (RCL) cleaved at the “P1” location. Acp62F's predicted P1 residue is at position 61 (Lys^61^) of the *D. melanogaster* protein, with the predicted RCL consisting of residues within approximately five sites on either side of P1. None of the sites for which positive selection is inferred lie within the RCL (Table 2), suggesting that direct interactions with the target protease(s) do not underlie selection on Acp62F. Rather, we hypothesize that the selected sites are involved in nonreactive site interactions with target proteases, or that changes at these residues alter the shape and/or reaction kinetics of the RCL.

Findlay et al. (2008) also documented evidence of positive selection on Acp62F as part of a large-scale survey of the molecular evolution of *Drosophila* seminal proteins identified via mass spectrometry, using sequence data from the same set of species used here. Our results extend Findlay et al.'s inference of positive selection using more sophisticated statistical models (the REL and FEL models of Kosakovsky Pond and Frost 2005), and further the analysis of the causes of selection on this protein.

### Experimental response to natural, but not sexual, selection

**Figure 1:**
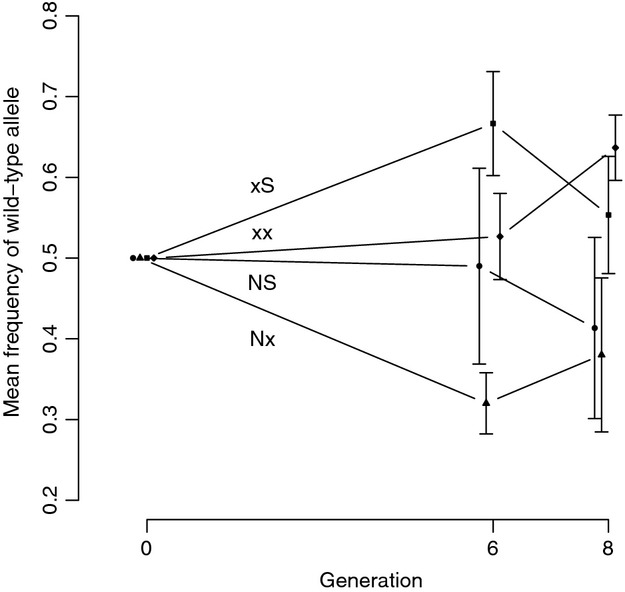
Changes in *Acp62F* wt allele frequency over time. Three populations were maintained under each combination of natural (N) and sexual (S) selection. Points represent the mean of the three populations, +/− 1 standard error.

While statistical analyses of sequence data indicate a history of positive selection on Acp62F, they provide little insight into the specific processes underlying selection. Several studies have examined the effects of Acp62F on phenotypes known to be mediated by *Drosophila* seminal proteins, but have failed to find a clear benefit to producing Acp62F. In particular, knockout mutants have *improved* sperm competitive ability (Mueller et al. 2008), and induced overexpression of Acp62F is toxic in preadult and adult flies (Lung et al. 2002). We therefore used an experimental strategy to infer the effects of Acp62F on net sexual and nonsexual fitness. We manipulated the opportunities for natural and sexual selection in a two-way factorial design, tracking the frequency of the *Acp62F* wt and null alleles. We found a strong effect of natural selection, with the null allele occurring at higher frequency in the presence than the absence of natural selection at both generation 6 and generation 8 (Fig. 1). For both treatments in which natural selection was unimpeded (Nx and NS), the frequency of the wt allele decreased over time relative to its starting frequency of 0.5, regardless of the presence or absence of sexual selection (NS and Nx, respectively, in Fig. 1). By contrast, for populations wherein natural selection was greatly reduced, the frequency of the wt allele was slightly elevated at generation 8 relative to its initial value. In a binomial regression, natural selection present/absent was a significant predictor of allele counts at both generation 6 and generation 8, with neither sexual selection present/absent nor their interaction being significant (Table 3).

**Table 3:** Effects of natural and sexual selection on *Acp62F* allele counts

	Generation 6			Generation 8		
		
Factor	Estimate	SE	*P*-value	Estimate	SE	*P*-value
Natural selection	-0.868	0.346	0.012	-1.035	0.322	0.001
Sexual selection	0.582	0.344	0.091	-0.285	0.321	0.374
N × S	0.117	0.489	0.811	0.401	0.450	0.373

Our failure to find an effect of *Acp62F* genotype on sexual fitness contrasts with results of a previous study examining the roles of Acp62F in various components of sexual and nonsexual fitness. Mueller et al. (2008) found that sperm from *Acp62F* null males were better able to resist displacement by a second male's sperm (i.e., P1 was higher for null males). Thus, it might have been expected that the *Acp62F* null allele would have increased in frequency in the sexual selection present treatments in our study, but this was not observed. Differences in experimental design between our study and that of Mueller et al. (2008) may help to explain these apparently contradictory results. Whereas Mueller et al. (2008) assayed a number of fitness components in isolation, the experimental evolution protocol used here integrates over all components of fitness, including female productivity, male mating success, and male sperm competitive ability. Thus, any sperm competitive advantage held by *Acp62F* null males in our study may have been offset by fitness deficits in other components of sexual fitness. Other authors have also reported differences in the fitness effects of individual mutations in assays of single-fitness components as compared with integrated measures of fitness (e.g., Arbuthnott and Rundle 2012).

Our finding that the *Acp62F* null allele is favored by natural selection, as well as a previous study finding a benefit for the null allele in sperm competition (Mueller et al. 2008), raises the question as to why a functional *Acp62F* gene persists in natural populations. There are at least two possible explanations. First, the laboratory environment may impose or relieve constraints present in natural populations, or otherwise alter conditions such that the effects of *Acp62F* genotype on laboratory fitness may not fully reflect the natural setting. For example, while remating was possible during our experiment, rates may have been lower than those in natural populations due to the truncated adult life span. Second, because we used an isogenic, rather than an outbred, background (as did Mueller et al. 2008), the results obtained here may not be fully representative of Acp62F's average fitness effect in an outbred population.

### Sexual selection neither reinforces nor inhibits natural selection on Acp62F

Our data are also relevant to the effects of sexual selection on nonsexual fitness, a topic of recent interest for which empirical data are mixed (Whitlock and Agrawal 2009). On the one hand, there are several ways by which sexual selection may reduce population mean fitness, including via the evolution of costly sexual displays and preferences for them, by generating sex-specific selection that leads to sexual conflict, and by reducing effective population size and thus increasing drift load. Alternatively, sexual selection may promote adaptation and the purging of deleterious alleles by inducing positive assortative mating for fitness, via a good genes process (i.e., the evolution of mate preferences for individuals of high breeding value for fitness), or more generally if reproductive success is condition- dependent (Whitlock and Agrawal 2009). Manipulating the opportunity for sexual selection and then tracking the frequency of individual alleles during experimental evolution provides a powerful and straightforward approach to comparing such costs and benefits. To our knowledge this approach has been applied in only two cases to date, both in *D. melanogaster*. One experiment used an alcohol dehydrogenase (*Adh*) null allele and provided results consistent with a benefit of sexual selection (Hollis et al. 2009); the other used six independent recessive mutations with visible phenotypic effects, and results suggested no benefit, and in some cases a cost, of sexual selection (Arbuthnott and Rundle 2012). Results of the current experiment using *Acp62F* provide another example of the latter, with no evidence that sexual selection favored the null allele with the highest nonsexual fitness. It is important to keep in mind, however, that *Acp62F* was not randomly chosen with respect to the potential alignment of natural and sexual selection, given an a priori expectation of ongoing sexual conflict (although no evidence suggesting such conflict was found).

## Appendix 2: Genotype counts and inferred allele frequencies at generations 6 and 8

**Table:**

Gen	Rep	Treatment	Hom_wt	Het	Hom_null	P	q	n
6	1	NS	0	6	6	0.25	0.75	12
6	1	Nx	0	9	3	0.38	0.63	12
6	1	xS	6	5	1	0.71	0.29	12
6	1	xx	5	4	3	0.58	0.42	12
6	2	NS	5	4	2	0.64	0.36	11
6	2	Nx	1	6	5	0.33	0.67	12
6	2	xS	7	4	1	0.75	0.25	12
6	2	xx	2	6	4	0.42	0.58	12
6	3	NS	5	4	3	0.58	0.42	12
6	3	Nx	0	6	6	0.25	0.75	12
6	3	xS	3	7	2	0.54	0.46	12
6	3	xx	4	6	2	0.58	0.42	12
8	1	NS	4	4	5	0.46	0.54	13
8	1	Nx	4	8	2	0.57	0.43	14
8	1	xS	5	7	3	0.57	0.43	15
8	1	xx	4	7	1	0.63	0.38	12
8	2	NS	1	4	10	0.20	0.80	15
8	2	Nx	1	7	7	0.30	0.70	15
8	2	xS	6	8	1	0.67	0.33	15
8	2	xx	6	5	4	0.57	0.43	15
8	3	NS	3	9	1	0.58	0.42	13
8	3	Nx	2	4	9	0.27	0.73	15
8	3	xS	1	8	3	0.42	0.58	12
8	3	xx	6	5	1	0.71	0.29	12
